# Predictors of Arterial Stiffness Amongst the 24-Hour Ambulatory Blood Pressure Variables in Hypertensive Patients

**DOI:** 10.7759/cureus.12207

**Published:** 2020-12-21

**Authors:** Vishal Bhandari, Kamal Sharma, Purva Shah, Erum Khan, Hardik D Desai, Tanisha Vora, Sukriti Bhalla, Dhruvkumar Gadhiya, Manish Bansal, Ravi R Kasliwal

**Affiliations:** 1 Interventional Cardiology, Tagore Hospital and Heart Care Center, Jalandhar, IND; 2 Cardiology Department, U.N. Mehta Institute of Cardiology and Research Centre, Ahmedabad, IND; 3 Medicine, B.J. Medical College, Ahmedabad, IND; 4 Internal Medicine, Gujarat Adani Institute of Medical Sciences, Krantiguru Shyamji Krishna Verma (KSKV) University, Bhuj, IND; 5 Graduate Medical Education, Smt. Nathiba Hargovandas Lakhmichand (NHL) Municipal Medical College, Ahmedabad, IND; 6 Cardiology, Akash Healthcare - Super Speciality Hospital, New Delhi, IND; 7 Cardiology, Medanta - The Medicity, Gurgaon, IND; 8 Cardiology, Medanta Hospital, Gurgaon, IND

**Keywords:** arterial stiffness, ambulatory blood pressure monitoring, high blood pressure, pwv, pulse wave velocity

## Abstract

Objective

The objective of the study is to identify the predominant determinants of arterial stiffness as assessed by pulse-wave-velocity (PVW) amongst various 24-hour ambulatory blood pressure monitoring (ABPM) parameters in Indian hypertensive subjects.

Method

Subjects of both genders between 18-60 years with hypertension and who were either drug naïve or on stable anti-hypertensive treatment for at least three months were included in the study. All subjects underwent clinical evaluation with a medical history, biochemical investigations, and assessment of arterial stiffness by PWV along with 24-hour ABPM.

Results

We found the males were younger than females amongst hypertensive cohort (41.53 ± 10.89 years vs. 52.2 ± 5.17 years, respectively; p=0.001) and had shorter duration of hypertension (41.42 ± 49.14 months vs. 87.8 ± 74.55 months, respectively; p=0.012) and had lower 24-hour average pulse pressure (aPP; 49.1 ± 7.8 mm Hg vs. 57.83 ± 8.92 mm Hg, respectively; p=0.001) at baseline. Younger people (<40-years) as compared to those >40-years of age had the lower carotid-femoral (cf) PWV (972.8 ± 125.0 cm/sec vs. 1165.0 ± 208.4 cm/sec, respectively; p=0.001) and average brachial-ankle (ba) PWV (1413.7 ± 160.4 cm/sec and 1640.0 ± 227.1 cm/sec, respectively; p=0.001). Bivariate analysis revealed that amongst all the 24-hour ABPM parameters, 24-hour aPP had the strongest correlation (r=0.414, p=0.003) with arterial stiffness as assessed by PWV. Also, statistically significant correlation was found in age group <40 years between cf-PWV and both 24-hour aPP (r=0.54, p=0.025) as well as night-time aPP (r=0.59, p=0.013)

Conclusion

We conclude that 24-hour aPP showed the strongest correlation with arterial stiffness parameters and best correlated with arterial stiffness variables amongst 24-hour ABPM parameters, especially amongst subjects <40 years of age. The pulsatile blood pressure (BP) was a better predictor of aortic PWV than the continuous part of BP.

## Introduction

Cardiovascular diseases are the leading cause of death worldwide, and now India is also synchronizing with global data. The major risk factor contributing to cardiovascular diseases is hypertension (HTN), which contributes to nearly 7.1 million premature deaths, two-thirds of all strokes, and half of all myocardial infarction every year [1].

It affected an estimated 118 million inhabitants in India in 2000; this number is projected to double to 214 million by 2025 [2]. HTN is directly responsible for 57% of all stroke deaths in India and 24% of all coronary heart disease (CHD) deaths in India [3]. Present estimates suggest that a 2 mm Hg population-wide decrease in systolic blood pressure (BP) can lead to the prevention of more than 151,000 strokes and 153,000 CHD deaths in India [4].

Recently ambulatory blood pressure monitoring (ABPM) is also gaining importance due to its benefits like measurement of the white coat effect, masked effect, and nocturnal dipping in hypertensives. Arterial stiffness is increasingly recognized as an important and independent contributor to cardiovascular (CV) morbidity and mortality in many patient subsets, particularly in the elderly, hypertensive, and those with end-stage renal disease [5-12]. Aortic stiffening causes loss of elastic recoil and affects the attenuation of the reflected wave, bringing changes in the systolic and diastolic blood pressure (amongst other parameters) in the peripheral and central vessels. Various techniques have been employed for the measurement of arterial stiffness in the central vessels through pulse wave analysis, like pulse wave velocity (PWV) of brachio-ankle and carotid-femoral and augmentation index, of which carotid-femoral PWV (cf-PWV) is a gold standard measure of aortic stiffness [5, 7]. 

Several studies have measured arterial stiffness in Indian subjects, but none has adequately reported its relationship with HTN by employing ambulatory BP monitoring [13-15]. This study was designed to identify the predominant determinant of arterial stiffness as assessed by PWV among different 24-hour ABPM parameters in Indian subjects.

## Materials and methods

Fifty-one subjects aged between 18 to 60 years presenting to the cardiology outpatient department from a period from January 2017 to December 2017 who were diagnosed to be a case of essential hypertension and who were either on no anti-hypertensive medication or on stable anti-hypertensives for the preceding three months or more were included in the study.

Subjects not willing to participate, diagnosed as a case of secondary hypertension, impaired renal functions, pregnant, with previously established cardiovascular disease, essential hypertension on medication for less than three months, and with a present history of any acute systemic illness were excluded from our study. We also excluded patients with diabetes mellitus or who had a history of tobacco consumption due to potential confounding effects of these factors on arterial stiffness. 

The study protocol was approved by the institutional Ethics Committee (IEC). Informed consent was obtained from all the participants prior to their enrollment in the study. Once enrolled, all subjects underwent clinical evaluation and biochemical investigations and assessment of arterial stiffness and ambulatory BP monitoring.

Statistical analyses were done using SPSS for Windows version 24.0 (IBM Inc., Armonk, USA). Comparisons between the groups were carried out using the independent Student t-test. Correlations among different arterial stiffness measurements were assessed using Pearson’s correlation coefficients. A p-value < 0.05 was considered statistically significant.

## Results

The baseline clinical characteristics of the study population are presented in Table 1. Subjects between the age of 18 to 60 years who were a diagnosed case of essential hypertension (n=51) and who fulfilled the inclusion and exclusion criteria of the study were included in the study. The mean age of all the subjects included in the study was 44.67 + 10.70, and 70.6 % of them were males.

**Table 1:** Baseline clinical characteristics of study subjects BMI - body mass index; SBP - systolic blood pressure; DBP - diastolic blood pressure; PP - pulse pressure; cf-PWP - carotid to femoral pulse wave velocity; baPWV - brachial-ankle pulse wave velocity; AIx - augmentation index; LDL - low-density lipoprotein; HDL - high-density lipoprotein; avg - average; HR - heart rate

Parameters	Mean +/- SD or n (%); n=51
Age (in years)	44.67 + 10.70
Gender	
Male	36 (70.6 %)
Female	15 (29.4 %)
Hypertension	51 (100 %)
Diabetes	0 (0 %)
Smoker	0 (0 %)
BMI (kg/m^2^)	28.71 + 4.65
Height (cm)	167.57 + 9.00
Weight (kg)	79.90 + 15.78
Clinic SBP (mm Hg)	142.04 + 14.41
Clinic DBP (mm Hg)	89.57 + 8.89
Ambulatory BP parameters	
24-hour avg. SBP (mm Hg)	138.35 + 13.46
24-hour avg. DBP (mm Hg)	86.68 + 9.33
Day-time avg. SBP (mm Hg)	141.55 + 13.05
Day-time avg. DBP (mm Hg)	89.33 + 9.05
Night-time avg. SBP (mm Hg)	130.74 + 15.54
Night-time avg. DBP (mm Hg)	79.05 + 12.34
24 hr. avg. PP (mm Hg)	51.67 + 9.00
Day-time avg. PP (mm Hg)	52.22 + 9.13
Night-time avg. PP (mm Hg)	51.70 + 8.73
Systolic nocturnal dipping (%)	-7.76 + 6.04
Diastolic nocturnal dipping (%)	-8.41 + 6.85
Pulse wave velocity parameters	
cf-PWV (cm/sec)	1100.98 + 205.05
avg. baPWV (cm/sec)	1564.57 + 232.12
AIx (%)	24.41 + 7.78
AIx (%) @ HR=75	24.20 + 9.31
Heart rate (bpm)	75.24 + 11.44
Total cholesterol (mg/dl)	162.78 + 35.23
LDL cholesterol (mg/dl)	102.61 + 29.19
HDL cholesterol (mg/dl)	41.25 + 9.60

Out of all the subjects (n=51), 29 subjects (56.1%) were on regular treatment with either single or a combination of anti-hypertensive medications for more than three months. The remaining 22 subjects (43.9 %) were not on any regular anti-hypertensive medication. The distribution of subjects according to BMI is as follows: the majority (94.11 %) were overweight (body-mass index > 23.0 kg/m^2^) with an overall mean body mass index of 28.71 kg/m^2^.

Our study observed that 51% of subjects had dyslipidemia, out of which only 11.76% of the total had high total cholesterol, and 45.1% had low levels of high-density lipoprotein (HDL) cholesterol.

The distribution of 24-hour ambulatory parameters is given in Table 1.

Arterial stiffness parameters

We analyzed various pulse wave velocity parameters (cf-PWV, average baPWV, augmentation index [Alx] %, and AIx % at heart rate [HR] = 75) during the study. We divided the subjects into two groups according to gender and compared the various parameters between the two groups. The results of the independent sample t-test for both males and females are presented in Table 2. Among all the parameters compared between males and females, we found the differences of age, duration of hypertension, 24-hour average pulse pressure, day-time average pulse pressure, night-time average pulse pressure, cf-PWV, and HDL cholesterol to be statistically significant.

**Table 2:** Results of the independent sample t-test for comparison of the mean value of various parameters between the two groups according to gender BMI - body mass index; SBP - systolic blood pressure; DBP - diastolic blood pressure; PP - pulse pressure; cf-PWP - carotid to femoral pulse wave velocity; baPWV - brachial-ankle pulse wave velocity; AIx - augmentation index; LDL - low-density lipoprotein; HDL - high-density lipoprotein; avg - average; HR - heart rate

Parameters	Male (n=36)	Female (n=15)	p-value
Age (years)	41.53 ± 10.89	52.2 ± 5.17	0.001
Duration of hypertension (months)	41.42 ± 49.14	87.8 ± 74.55	0.012
BMI (kg/m2)	28.62 ± 4.76	28.91 ± 4.5	0.843
HR (bpm)	74.94 ± 11.44	75.93 ± 11.79	0.782
Clinic SBP (mm Hg)	140.83 ± 12.72	144.93 ± 18.03	0.360
Clinic DBP (mm Hg)	90.33 ± 8.69	87.73 ± 9.41	0.347
24-hour avg. SBP (mm Hg)	136.06 ± 11.81	143.83 ± 15.9	0.059
24-hour avg. DBP (mm Hg)	86.96 ± 8.8	86.01 ± 10.81	0.744
Day-time avg. SBP (mm Hg)	139.6 ± 11.45	146.23 ± 15.73	0.099
Day-time avg. DBP (mm Hg)	89.85 ± 8.4	88.09 ± 10.66	0.531
Night-time avg. SBP (mm Hg)	128.16 ± 13.48	136.93 ± 18.71	0.066
Night-time avg. DBP (mm Hg)	78.59 ± 12.05	80.15 ± 13.39	0.685
24-hour avg. PP (mm Hg)	49.1 ± 7.8	57.83 ± 8.92	0.001
Day-time avg. PP (mm Hg)	49.75 ± 7.84	58.14 ± 9.53	0.002
Night-time avg. PP (mm Hg)	49.58 ± 8.05	56.78 ± 8.43	0.006
Systolic nocturnal dipping (%)	-8.19 ± 5.96	-6.73 ± 6.3	0.435
Diastolic nocturnal dipping (%)	-9.28 ± 6.95	-6.33 ± 6.32	0.163
cf-PWV (cm/sec)	1064.15 ± 195.14	1189.35 ± 207.60	0.046
avg. baPWV (cm/sec)	1533.1 ± 229.2	1640.1 ± 228.9	0.135
AIx (%)	23.75 ± 6.84	26 ± 9.75	0.352
AIx (%) @ HR=75	23.5 ± 8.79	25.87 ± 10.59	0.414
Total cholesterol (mg/dl)	160.72 ± 38.61	167.73 ± 25.85	0.523
LDL cholesterol (mg/dl)	102.75 ± 31.71	102.27 ± 23	0.958
HDL cholesterol (mg/dl)	39 ± 9	46.67 ± 9.07	0.008

We analyzed the subjects into two groups according to age (i.e., age <40 years and >40 years) and compared the various parameters between the two groups. The results of the independent sample t-test for both the age groups are presented in Table 3. Among all the parameters compared between the two groups, we found the differences between cf-PWV, average baPWV, AIx (%), AIx (%) @ HR=75, day-time average pulse pressure, and height to be statistically significant.

**Table 3:** Results of the independent sample t-test for comparison of the mean value of various parameters between the two groups according to age BMI - body mass index; SBP - systolic blood pressure; DBP - diastolic blood pressure; PP - pulse pressure; cf-PWP - carotid to femoral pulse wave velocity; baPWV - brachial-ankle pulse wave velocity; Alx - augmentation index; LDL - low-density lipoprotein; HDL - high-density lipoprotein; avg - average; HR - heart rate

Parameters	Age groups				p-value
	<40 years		>40 years		
	Mean	Standard deviation	Mean	Standard deviation	
Height (cms)	175	6	164	8	<0.001
BMI (kg/m2)	28.6	5.1	28.8	4.5	0.895
HR (bpm)	78	11	74	11	0.188
Total cholesterol (mg/dl)	162	35	163	36	0.925
LDL cholesterol (mg/dl)	105	26	101	31	0.661
HDL cholesterol (mg/dl)	37	9	43	9	0.036
cf-PWV (cm/sec)	972.8	125.0	1165.0	208.4	0.001
avg. baPWV (cm/sec)	1413.7	160.4	1640.0	227.1	0.001
AIx (%)	20.4	6.1	26.4	7.8	0.008
AIx (%) @ HR=75	19.1	7.0	26.8	9.3	0.004
24-hour avg SBP (mm Hg)	134.0	9.9	140.5	14.6	0.103
Day-time avg. SBP (mm Hg)	137.1	10.1	143.8	13.9	0.083
Night-time avg. SBP (mm Hg)	126.4	11.6	132.9	16.9	0.158
24-hour avg. PP (mm Hg)	48.7	7.5	53.5	9.3	0.069
Day-time avg. PP (mm Hg)	48.0	7.5	54.7	9.3	0.012
Night-time avg. PP (mm Hg)	50.9	8.7	52.6	9.2	0.514
Systolic nocturnal dipping (%)	-7.7	7.1	-7.8	5.5	0.956
24-hour avg. DBP (mm Hg)	85.92	9.47	87.05	9.38	0.688
Day-time avg. DBP (mm Hg)	89.23	9.62	89.39	8.90	0.954
Night-time avg. DBP (mm Hg)	76.4	11.3	80.3	12.8	0.285

Pearson’s correlation coefficients (r) between 24-hour ambulatory blood pressure parameters and pulse wave velocity parameters are presented in Table 4. Bivariate analysis revealed that 24-hour average pulse pressure, day-time average pulse pressure, night-time average pulse pressure, 24-hour average systolic blood pressure (SBP), day-time average SBP, and age were statistically significantly correlated with cf-PWV. Among all the 24-hour ambulatory blood pressure parameters, 24-hour average pulse pressure was observed to have the strongest correlation (r=0.414, p=0.003) with arterial stiffness.

**Table 4:** Pearson’s correlation coefficient between 24-hour ambulatory blood pressure parameters and pulse wave velocity parameters SBP - systolic blood pressure; DBP - diastolic blood pressure; PP - pulse pressure; cf-PWP - carotid to femoral pulse wave velocity; baPWV - brachial-ankle pulse wave velocity; AIx - augmentation index; avg - average; HR - heart rate ** Correlation is significant at the 0.01 level (2-tailed). * Correlation is significant at the 0.05 level (2-tailed).

Parameters		cf-PWV	avg. baPWV	AIx (%)	AIx (%) @ HR=75
24-hour avg. SBP (mm Hg)	Pearson correlation	0.339^*^	0.324^*^	0.295^*^	0.338^*^
	p-value	0.015	0.02	0.035	0.015
24-hour avg. DBP (mm Hg)	Pearson Correlation	0.094	0.164	0.08	0.078
	p-value	0.512	0.249	0.576	0.588
Day-time avg. SBP (mm Hg)	Pearson correlation	0.350^*^	0.338^*^	0.317^*^	0.331^*^
	p-value	0.012	0.015	0.023	0.018
Day-time avg. DBP (mm Hg)	Pearson correlation	0.088	0.164	0.071	0.05
	p-value	0.538	0.25	0.62	0.727
Night-time avg. SBP (mm Hg)	Pearson correlation	0.261	0.246	0.254	0.292^*^
	p-value	0.064	0.081	0.073	0.038
Night-time avg. DBP (mm Hg)	Pearson correlation	0.077	0.118	0.077	0.102
	p-value	0.59	0.408	0.59	0.474
24-hour avg. PP (mm Hg)	Pearson correlation	0.414^**^	0.322^*^	0.372^**^	0.425^**^
	p-value	0.003	0.021	0.007	0.002
Day-time avg. PP (mm Hg)	Pearson correlation	0.397^**^	0.309^*^	0.377^**^	0.420^**^
	p-value	0.004	0.027	0.006	0.002
Night-time avg. PP (mm Hg)	Pearson correlation	0.335^*^	0.195	0.288^*^	0.326^*^
	p-value	0.016	0.17	0.04	0.019
Systolic nocturnal dipping (%)	Pearson correlation	-0.012	-0.03	0.021	0.069
	p-value	0.931	0.833	0.884	0.63

We found a statistically non-significant correlation (r=-0.012, p=0.931) between systolic nocturnal dipping and cf-PWV. Pearson’s correlation coefficient between pulse wave velocity and other clinical parameters is presented in Table 5.

**Table 5:** Pearson’s correlation coefficient between pulse wave velocity parameters and clinical parameters BMI - body mass index; cf-PWP - carotid to femoral pulse wave velocity; baPWV - brachial-ankle pulse wave velocity; Alx - augmentation index; LDL - low-density lipoprotein; HDL - high-density lipoprotein; avg - average; HR - heart rate ** Correlation is significant at the 0.01 level (2-tailed). * Correlation is significant at the 0.05 level (2-tailed).

Parameters		cf-PWV	avg. baPWV	AIx (%)	AIx (%) @ HR=75
Age	Pearson correlation	0.357^*^	0.444^**^	0.330^*^	0.382^**^
	p-value	0.010	0.001	0.018	0.006
Height (cms)	Pearson correlation	-0.356^*^	-0.348^*^	-0.182	-0.158
	p-value	0.010	0.012	0.202	0.269
BMI (kg/m2)	Pearson correlation	-0.105	-0.158	-0.173	-0.171
	p-value	0.463	0.269	0.226	0.231
HR (bpm)	Pearson correlation	-0.079	-0.089	-0.069	-0.508^**^
	p-value	0.580	0.534	0.632	0.000
Total cholesterol (mg/dl)	Pearson correlation	0.113	0.155	0.018	0.083
	p-value	0.430	0.276	0.902	0.562
LDL cholesterol (mg/dl)	Pearson correlation	0.011	-0.016	-0.117	-0.051
	p-value	0.939	0.911	0.414	0.725
HDL cholesterol (mg/dl)	Pearson correlation	0.245	0.366^**^	0.281^*^	0.329^*^
	p-value	0.084	0.008	0.046	0.018

Pearson’s correlation coefficient between pulse wave velocity and 24-hour ambulatory blood pressure parameters in the age group <40 years and >40 years is presented in Table 6. We found statistically significant correlation in the age group <40 years between cf-PWV and both 24-hour average pulse pressure (r=0.54, p=0.025) and night-time pulse pressure (r=0.59, p=0.013), respectively.

**Table 6:** Pearson’s correlation coefficient between 24-hour ambulatory blood pressure parameter and pulse wave velocity parameters in different age groups SBP - systolic blood pressure; DBP - diastolic blood pressure; PP - pulse pressure; cf-PWP - carotid to femoral pulse wave velocity; baPWV - brachial-ankle pulse wave velocity; AIx - augmentation index; avg - average; HR - heart rate ** Correlation is significant at the 0.01 level (2-tailed). * Correlation is significant at the 0.05 level (2-tailed).

Parameters		cf-PWV		avg. baPWV		AIx (%)		AIx (%) @ HR=75	
		Age ≤40 years (n=17)	Age >40 years (n=34)	Age ≤40 years (n=17)	Age >40 years (n=34)	Age ≤40 years (n=17)	Age >40 years (n=34)	Age ≤40 years (n=17)	Age >40 years (n=34)
24-hour avg. SBP	Pearson correlation	0.384	0.249	0.469	0.201	0.344	0.204	0.265	0.279
	p-value	0.128	0.155	0.057	0.255	0.177	0.247	0.304	0.110
24-hour avg. DBP	Pearson correlation	0.050	0.087	0.350	0.091	0.070	0.062	0.150	0.028
	p-value	0.850	0.626	0.168	0.609	0.791	0.729	0.565	0.876
Day-time avg. SBP	Pearson correlation	0.311	0.271	0.434	0.218	0.296	0.240	0.160	0.290
	p-value	0.225	0.121	0.082	0.216	0.248	0.172	0.540	0.096
Day-time avg. DBP	Pearson correlation	-0.009	0.131	0.334	0.129	0.048	0.085	0.085	0.038
	p-value	0.973	0.460	0.190	0.468	0.854	0.634	0.744	0.829
Night-time avg. SBP	Pearson correlation	0.473	0.141	0.458	0.110	0.452	0.131	0.418	0.191
	p-value	0.055	0.426	0.064	0.534	0.069	0.459	0.095	0.278
Night-time avg. DBP	Pearson correlation	0.107	-0.014	0.244	-0.002	0.063	0.010	0.199	-0.002
	p-value	0.683	0.937	0.346	0.989	0.811	0.957	0.444	0.993
24-hour avg. PP	Pearson correlation	0.540^*^	0.303	0.284	0.224	0.472	0.259	0.209	0.410^*^
	p-value	0.025	0.081	0.270	0.203	0.056	0.139	0.420	0.016
Day-time avg. PP	Pearson correlation	0.419	0.259	0.149	0.186	0.320	0.275	0.097	0.396^*^
	p-value	0.094	0.139	0.567	0.292	0.210	0.116	0.710	0.020
Night-time avg. PP	Pearson correlation	0.590^*^	0.265	0.163	0.177	0.421	0.222	0.279	0.332
	p-value	0.013	0.129	0.531	0.318	0.092	0.206	0.278	0.055
Systolic nocturnal dipping (%)	Pearson correlation	0.267	-0.114	0.130	-0.102	0.252	-0.084	0.331	-0.035
	p-value	0.300	0.521	0.618	0.565	0.328	0.636	0.194	0.845

## Discussion

Our study is the first study amongst Asian Indians to report the relationship between various 24-hour ambulatory blood pressure parameters and arterial stiffness in middle-aged hypertensive subjects. The results of our studies show:

1) arterial stiffness increases progressively as BP increases;

2) 24-hour average pulse pressure, day-time average pulse pressure, night-time average pulse pressure, 24-hour average SBP, day-time average SBP, and age were statistically significantly correlated with cf-PWV, the strongest correlation being with 24-hour average pulse pressure;

3) there was no significant correlation between ‘nocturnal dipping’ and arterial stiffness;

4) in the age group <40 years, we found a statistically significant correlation between cf-PWV and both 24-hour average pulse pressure and night-time pulse pressure;

5) the pulsatile BP had more impact on aortic PWV than the continuous part of BP.

Arterial stiffness is related to BP and is an independent predictor of CV events in hypertensives, so it is recommended in evaluation [16]. Ngim et al. and Stompor et al. reported that cf-PWV was correlated with SBP and also mean arterial pressure (MAP), but not with diastolic blood pressure (DBP) in untreated hypertensive and normotensive middle-aged Malay men and in peritoneal dialysis patients, respectively [16, 17]. Those findings are consistent with our results. Tingli Qin et al. concluded that in patients with grade 1/grade 2 essential hypertension, ambulatory arterial stiffness index (AASI) shows a significant correlation with ambulatory pulse pressure, which is quite similar to our study results [18].

Sa Cunha et al. recommended gender difference - in both sexes, SBP showed a correlation with PWV, while only in women, DBP was correlated with PWV [19]. In contrast, Nurnberger et al. reported that DBP was the only important hemodynamic determinant of PWV in young healthy males [20]. The current study may be confounded by different demographic characteristics such as age range, gender distribution, and BMI than previous studies apart from the smaller sample size. The antihypertensive agents used also might affect the results [21]. Besides, the various methods of BP measurement (e.g., 24-hour ambulatory BP monitoring, casual BP measurement, automatic BP monitoring for 30 minutes) could attribute to the results [22]. Since our findings also revealed the prognostic importance of SBP and PP for aortic PWV and no clear association between DBP and PWV, the results were consistent with previous evidence [23-25]. Aortic PWV has been described as a superior independent indicator of cardiovascular outcome in population-based trials, even after modification of conventional cardiovascular risk factors [26-27]. Elevated PP also has been known to be an independent risk factor of cardiovascular disease [26-30].

Interestingly, Nurnberger et al. reported a contrary result [20]. They showed that among all BP parameters, DBP was the only significant determinant of PWV. But only young (23-35 years of age) healthy males were included in the sample group, where DBP was considered to be the best indicator of coronary heart disease in the Framingham heart study. While the findings of the current study and the Nurnberger study are different, they can jointly reflect the different age-related relationship between BP parameters and PWV. Aortic PWV and BP are proposed to be highly affected by age, and the function of BP parameters as a PWV predictor can vary according to the age spectrum of the population studied.

In a similar study by Kim et al., authors measured BP by invasive measures and also found that among a number of BP parameters, PP demonstrated the greatest association with aortic PWV in normotensive and untreated middle-aged and elderly hypertensive subjects [30]. As the aorta and its first branches are responsible for much of the pathophysiological consequences of arterial stiffness, PWV calculated in the aortic and aortofemoral routes has been recognized to be the most clinically important.

Our study is the first Indian study to report the relationship between various 24-hour ambulatory blood pressure parameters and arterial stiffness in middle-aged hypertensive subjects.

One of the limitations of our study was that although the non-invasive technique showed acceptable reproducibility, the length of the arterial segment was usually estimated by direct superficial measurement of the distance between two transducers. As arteries get longer and more tortuous with age, aortic PWV will therefore be overlooked by a non-invasive procedure. The restricted sample size was also another drawback of the analysis.

## Conclusions

This study concluded that in hypertensive middle-aged subjects, 24-hour average pulse pressure showed the strongest correlation with arterial stiffness and was the predominant determinant of arterial stiffness among different 24-hour ambulatory blood pressure parameters. Pulsatile BP had more impact on aortic PWV than the continuous part of BP. In combination with the previous studies, the present study also suggests that the role of ambulatory BP parameters as a predictor of PWV could be different according to the age range of the population studied.

## References

[1] Kearney P, Whelton M, Reynolds K (2005). Global burden of hypertension: analysis of worldwide data. Lancet.

[2] Gupta R (2004). Trends in hypertension epidemiology in India. J Hum Hyperens.

[3] Patel V, Chatterji S, Chisholm D (2011). Chronic diseases and injuries in India. Lancet.

[4] Laurent S, Cockcroft J, van Bortel L (2006). Expert consensus document on arterial stiffness: methodological issues and clinical applications. Eur Heart J.

[5] Meaume S, Benetos A, Henry O, Rudnichi A, Safar ME (2001). Aortic pulse wave velocity predicts cardiovascular mortality in subjects >70 years of age. Arterioscler Thromb Vasc Biol.

[6] Sutton-Tyrrell K, Najjar S, Boudreau R (2005). Elevated aortic pulse wave velocity, a marker of arterial stiffness, predicts cardiovascular events in well functioning older adults. Circulation.

[7] Boutouyrie P, Tropeano A, Asmar R (2002). Aortic stiffness is an independent predictor of primary coronary events in hypertensive patients: a longitudinal study. Hypertension.

[8] Laurent S, Boutouyrie P, Asmar R (2001). Aortic stiffness is an independent predictor of all-cause and cardiovascular mortality in hypertensive patients. Hypertension.

[9] Laurent S, Katsahian S, Fassot C (2003). Aortic stiffness is an independent predictor of fatal stroke in essential hypertension. Stroke.

[10] Blacher J, Guerin A, Pannier B (1999). Impact of aortic stiffness on survival in end-stage renal disease. Circulation.

[11] Vlachopoulos C, Aznaouridis K, Stefanadis C (2010). Prediction of cardiovascular events and all-cause mortality with arterial stiffness: a systematic review and meta-analysis. J Am Coll Cardiol.

[12] Milan A, Tosello F, Fabbri A (2011). Arterial stiffness: from physiology to clinical implications. High Blood Press Cardiovasc Prev.

[13] Kasliwal R, Bansal M, Bhargava K (2004). Carotid intima-media thickness and brachial-ankle pulse wave velocity in patients with and without coronary artery disease. Indian Heart J.

[14] Mohan V, Gokulakrishnan K, Ganesan A, Kumar SB (2010). Association of Indian diabetes risk score with arterial stiffness in Asian Indian non-diabetic subjects: the Chennai urban rural epidemiology study (CURES-84). J Diabetes Sci Technol.

[15] Mancia G, De Backer G, Dominiczak A (2007). 2007 Guidelines for the management of arterial hypertension: The Task Force for the Management of Arterial Hypertension of the European Society of Hypertension (ESH) and of the European Society of Cardiology (ESC). Eur Heart J.

[16] Ngim C, Rahman A, Ibrahim A (1999). Pulse wave velocity as an index of arterial stiffness: a comparison between newly diagnosed (untreated) hypertensive and normotensive middle-aged Malay men and its relationship with fasting insulin. Acta Cardiol.

[17] Stompor T, Rajzer M, Sulowicz W (2003). An association between aortic pulse wave velocity, blood pressure and chronic inflammation in ESRD patients on peritoneal dialysis. Int J Artif Organs.

[18] Qin T, Jiang H, Jiao Y (2014). Ambulatory arterial stiffness index correlates with ambulatory pulse pressure but not dipping status in patients with grade 1/grade 2 essential hypertension. J Int Med Res.

[19] Sa Cunha R, Pannier B, Benetos A (1997). Association between high heart rate and high arterial rigidity in normotensive and hypertensive subjects. J Hypertens.

[20] Nurnberger J, Dammer S, Opazo Saez A, Philipp T, Schäfers RF (2003). Diastolic blood pressure is an important determinant of augmentation index and pulse wave velocity in young, healthy males. J Hum Hypertens.

[21] Asmar R, Brunel P, Pannier B, Lacolley PJ, Safar ME (1988). Arterial distensibility and ambulatory blood pressure monitoring in essential hypertension. Am J Cardiol.

[22] Franklin S, Gustin W, Wong N (1997). Hemodynamic patterns of age-related changes in blood pressure. The Framingham heart study. Circulation.

[23] Khattar R, Swales J, Dore C, Senior R, Lahiri A (2001). Effect of aging on the prognostic significance of ambulatory systolic, diastolic, and pulse pressure in essential hypertension. Circulation.

[24] Franklin S, Larson M, Khan S (2001). Does the relation of blood pressure to coronary heart disease risk change with aging? The Framingham heart study. Circulation.

[25] Amar J, Ruidavets J, Chamontin B, Drouet L, Ferrières J (2001). Arterial stiffness and cardiovascular risk factors in a population-based study. J Hypertens.

[26] Willum-Hansen T, Staessen J, Torp-Pedersen C (2006). Prognostic value of aortic pulse wave velocity as index of arterial stiffness in the general population. Circulation.

[27] de Simone G, Roman M, Koren M (1999). Stroke volume/pulse pressure ratio and cardiovascular risk in arterial hypertension. Hypertension.

[28] Benetos A, Safar M, Rudnichi A (1997). Pulse pressure: a predictor of long-term cardiovascular mortality in a French male population. Hypertension.

[29] Verdecchia P, Schillaci G, Borgioni C (1998). Ambulatory pulse pressure: a potent predictor of total cardiovascular risk in hypertension. Hypertension.

[30] Kim E, Park C, Park J (2007). Relationship between blood pressure parameters and pulse wave velocity in normotensive and hypertensive subjects: invasive study. J Hum Hypertens.

